# Displacement correlations between a single mesenchymal-like cell and its nucleus effectively link subcellular activities and motility in cell migration analysis

**DOI:** 10.1038/srep34047

**Published:** 2016-09-27

**Authors:** Tian Lan, Kai Cheng, Tina Ren, Stephen Hugo Arce, Yiider Tseng

**Affiliations:** 1 Department of Chemical Engineering, Gainesville, FL 32611, USA; 2 Harvard School of Dental Medicine, Boston, MA 02115, USA; 3 J. Crayton Pruitt Family Department of Biomedical Engineering, Gainesville, FL 32611, USA; 4 Institute for Cell & Tissue Science and Engineering, University of Florida, Gainesville, FL 32611, USA; 5 National Cancer Institute-Physical Science Oncology Center, Gainesville, FL 32611, USA

## Abstract

Cell migration is an essential process in organism development and physiological maintenance. Although current methods permit accurate comparisons of the effects of molecular manipulations and drug applications on cell motility, effects of alterations in subcellular activities on motility cannot be fully elucidated from those methods. Here, we develop a strategy termed c*ell-nuclear* (*CN) correlation* to parameterize represented dynamic subcellular activities and to quantify their contributions in mesenchymal-like migration. Based on the biophysical meaning of the *CN correlation*, we propose a cell migration potential index (*CMPI*) to measure cell motility. When the effectiveness of *CMPI* was evaluated with respect to one of the most popular cell migration analysis methods, Persistent Random Walk, we found that the cell motility estimates among six cell lines used in this study were highly consistent between these two approaches. Further evaluations indicated that *CMPI* can be determined using a shorter time period and smaller cell sample size, and it possesses excellent reliability and applicability, even in the presence of a wide range of noise, as might be generated from individual imaging acquisition systems. The novel approach outlined here introduces a robust strategy through an analysis of subcellular locomotion activities for single cell migration assessment.

Cell migration is a highly coordinated event that plays a central role in a broad range of physiological and pathological events, including embryonic development^1^,^2^, wound healing^3^,^4^,^5^ and cancer metastasis^6^,^7^,^8^,^9^. During organogenesis, cell migration is critical to sustaining the functions of organ systems. Failure of cell migration to the correct positions can have grave consequences. For example, defective cell migration of the cardiac neural crest cells impairs aorticopulmonary septation in Splotch mutant mice and is often fatal^10^. Clinically, cell migration is particularly relevant as among cancer patients, the 5-year mortality rate is dramatically increased if cancer cells become metastatic, *i.e*., inappropriately escaping from their original location and migrating to distal sites^9^,^11^. Hence, the ability to precisely assess cell migration states could provide valuable insight into cell physiology and pathology.

Currently, the primary tools for quantitatively assessing the cell migration capacity are the wound-healing assay and single cell trajectory analysis. The wound-healing assay mimics the healing process of epithelial tissue^12^,^13^. It measures the ability of a monolayer of confluent cells to migrate into and fully cover a denuded area (a wound), which is created by scraping cells out of the monolayer. Effects of disruptive factors such as extracellular stimuli (drugs) or intracellular alterations (protein expression or activity changes) can be assessed by measuring their capacity to modulate the rate of confluence restoration^14^,^15^. Cell trajectory analysis of single mesenchymal-like cells is performed through using mathematical models, mainly persistent random walk (*PRW*)^16^. In this model, cell speed and average persistence time of duration (*i.e*., persistence time) as a cell moves towards or away from a stimulus are the critical parameters of cell motility.

These two approaches treat cells as a simple, uniform object and deal only with the positions and the trajectories of the cells^12^,^14^,^16^,^17^. Hence, variations among cells in migration strategies that result in different inherent migration capacities cannot be revealed directly from these approaches. In mesenchymal-like cells, cell migration involves complicated subcellular processes^18^,^19^,^20^. However, all these processes can be classified into two common, independent subcellular activities. The first is the formation of lamellipodia and filopodia in the cell leading edge, which enables a cell to protrude and establish new contacts with its environment^21^. The second is actomyosin contraction, which induces tension through stress fibers and induces the detachment of contact points at the trailing edge of the cell and drags the cell body toward the leading edge^22^. The detailed molecular mechanisms of these two subcellular activities have been elucidated^23^,^24^,^25^,^26^,^27^; yet, this knowledge has not resulted in a complete understanding of the relationship between mesenchymal-like cell migration patterns and cell motility.

Here, we report a new approach to evaluating cell motility through the contributions of these two common subcellular activities. The nucleus is tightly connected to the cytoskeleton^28^ and is responsive to cell movement^29^; therefore, it is possible to assess the cell migration status by analyzing the motion of the cell relative to the nucleus. We correlated individual cell centroid displacements with nuclear centroid displacements to identify real-time subcellular activities during cell migration. Consequently, we evaluated mesenchymal-like cell motility by concurrent analysis of the contributions of these two subcellular activities. Through cell movement analysis, this work provides a biological and physical connection between migration potential and molecular mechanisms of mesenchymal-like cell migration.

## Results

### CN correlations

Mesenchymal-like cell migration is a discrete process that can be classified into two main types of independent subcellular events: leading edge protrusions and trailing edge detachments. In a mesenchymal-like cell, the nucleus is generally located at the trailing edge. When a cell extends its leading edge to probe the environment, the nucleus remains stationary. Once the cell establishes new contacts and determines where to move, actomyosin contractions occur in the trailing edge; local adhesion sites are detached and the nucleus moves coherently with the main body of the cell. Since leading edge protrusions and trailing-edge detachments are distinguishable by the relative motion between momentary cell centroid displacement (*CCD*) and coupled nucleus centroid displacement (*NCD*). The relation between the *CCD* and the *NCD* might be applied to analyze cell migration.

**Figure 1:**
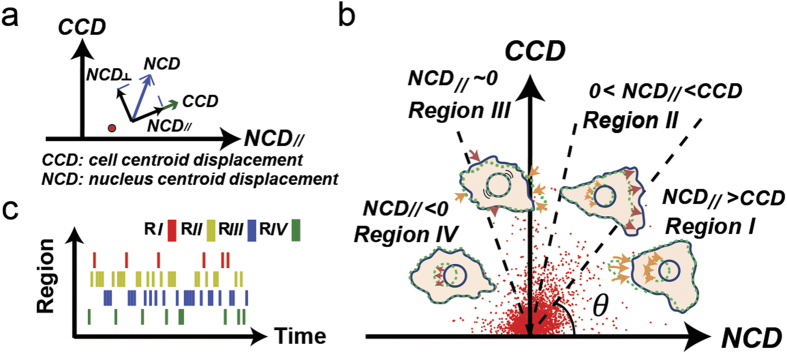
Correlations between cell and nucleus displacements can describe subcellular activities of cell migration. **(a)** The *CN correlation* data point (red dot) locates the correlation between a *CCD* and a coupled *NCD*_//_ (projection of nucleus centroid displacement in the *CCD* direction) in a *CCD vs. NCD*_//_ plot. **(b)** Four separate regions (separated by dash lines at 45°, 75°, and 105°) in the *CCD*-*NCD*_//_ coordinate system correspond to distinct subcellular migratory activities. A cluster of red dots shown are the *CN correlation* data acquired from 50 NIH 3T3 fibroblasts tracked at 1-min. time intervals for 1 hour. **(c)** The barcode enables visualization of the sequential occurrence of *CN correlations* in the four regions. Regions are distinguished by red, yellow, blue, and green, respectively. Each bar represents a *CN correlation* data point acquired at a 1-min time interval.

When a correlation exists between a *CCD* and coupled *NCD*, this correlation should share a common driving force. Hence, when the correlation is analyzed, the transient direction of every *CCD* could be set as the reference direction to correlate to the coupled *NCD* projected in the same direction (denoted as *NCD*_//_) (Fig. 1a). Based on the mesenchymal migratory mechanism, a trailing edge detachment event should yield a significant *NCD*_//_ due to the coherent movements between the cell body and the nucleus. In contrast, in a leading edge protrusion event, the *NCD*_//_ should be negligible because during the event the nucleus remains relatively stationary. We term the correlation between a *CCD* and the concomitant *NCD*_//_ a *CN correlation* and describe the migration status of a cell through a collection of *CN correlations*.

### *CN correlation* analysis is a new approach to elucidate the subcellular activities of cell migration

We hypothesize that a *CCD*-*NCD*_//_ coordinate system combined with the *CN correlation* approach can be used to describe the subcellular events of cell migration. The location of a *CN correlation* data point can be expressed using the polar coordinate system and a range of coordinate angles are used to delineate corresponding subcellular events (Fig. 1b).

During a pure protrusion or retraction event (we will use protrusion to represent both events below), the leading edge of the cell either extends to probe the environment or retracts back from a protrusion event if the anchorage to the ECM cannot be established while the nucleus remains immobile, *i.e*., *NCD*_// _~ 0. Therefore, the *CN correlation* should be located within an angular zone around 90° in the polar coordinates of the *CCD vs. NCD*_*//*_ plot. On the other hand, in a pure detachment event, the nucleus has roughly the same amount of coherent translocation as the trailing edge does; however, the relatively greater cell area in respect to the smaller nuclear area makes *NCD*_//_ > *CCD*. Thus, a detachment event would be found in the angular zone below 45° of the plot. In addition, the leading edge protrusion and the trailing edge detachment can occur and be documented simultaneously within a monitored time interval. Hence, both detachment and protrusion events contribute to the *CCD*. When the protrusion event is significant to a certain level, the *CCD* value contributed from the protrusion event could make the overall *CCD* value greater than the *NCD*_//_ value. This extra *CCD* would locate the corresponding *CN correlation* data point to the evasive migration zone, where it is in between the pure detachment zone and the pure protrusion zone. Moreover, a significantly negative *NCD*_//_ (where the *CCD* and the coupled *NCD* form an obtuse angle) might imply a considerable turning event. The cell could markedly alter its *CCD* direction (more than 90°) from the previous one when a large size protrusion forms, whereas the nucleus retains its inertia in the previous direction. Hence, the *NCD*_//_ could temporarily possess an opposite direction to the newly formed protrusion, resulting in a negative *CN correlation*.

These algorithms could provide an effective guideline to distinguish different types of subcellular activities of cell migration. Yet, this *CN correlation* concept does not come from a blind test. Each time, we need to monitor almost a complete cell locomotion event before we can determine the corresponding *CCD* and *NCD* for the analysis. However, each cell locomotion event takes different time to complete. Therefore, describing cell locomotion events using a fixed time lag cannot be achieved. Since a cell locomotion needs to be identified first before its duration can be determined, the *CN correlation* analysis can be validated only if it is independent from the durations of the locomotion events.

We therefore divide the entire cell motion into a series of motion fragments with fixed time intervals (*e.g*., 1 min). In this case, each locomotion during the whole cell motion period is also divided into a series of sub-locomotion events (subevents) with the same fixed time intervals. The subevents of a pure protrusion should possess the signature of the original protrusion, *i.e*., *NCD*_//_ ~ 0. Similarly, a pure detachment should propagate its signature, *NCD*_//_ > *CCD*, into its subevents. Shorter time intervals will also dissect an evasive migration event into a combination of subevents mixed with the signatures of pure protrusion and pure detachment events, and might be smaller evasive events. Finally, a large-angle turn can be well composed of evasive migration and large-angle protrusion, but must include some subevents with significantly negative *NCD*_//_ as a signature. Thus, entire cell motions can be displayed as a collection of continuous, independent subevents from shorter, fixed time intervals. All the locomotion events could also be identified through the *CN correlation* analysis.

In essence, subcellular migratory activities might be simply composed of a series of subevents, classified into four independent modes in a *CCD-NCD*_//_ coordinate system according to the corresponding *CN correlation* location — Region *I* (0° < *θ* ≤ 45°): *Detachment Mode*; Region *II* (45° < *θ* ≤ 75°): *Mixture of Detachment and Protrusion Mode*; Region *III* (75° < *θ* ≤ 105°): *Protrusion/Retraction Mode*; and Region *IV* (105° < *θ* ≤180°): *Large-angle Side Protrusion Mode* (Fig. 1b).

### Barcodes demonstrate real time cell migration dynamics

**Figure 2:**
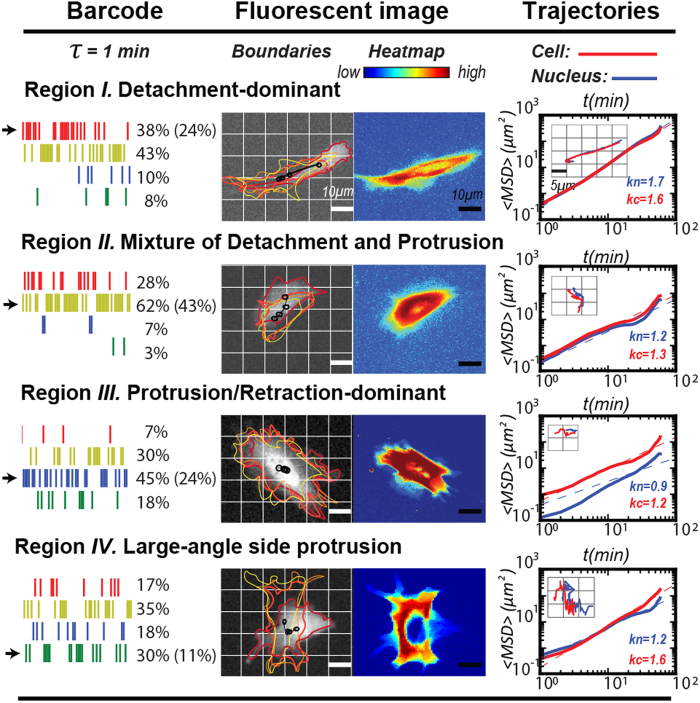
Specific barcodes identify significant locomotion events during the cell migration. In each row, a barcode, fluorescent image, and the trajectories of the cell and its nucleus are depicted in the left, middle, and right panels, respectively. *Left:* Each barcode, which displays the analysis results from its cell migration movie, was obtained from 1-min time intervals and possesses a dominant *CCD-NCD*_//_ relationship in one of the four regions of the *CN correlation*. The percentage of the individual bars in the barcode representing each region is showed in the right column. The averaged occurrence of a region overall (50 barcodes) is shown inside the parenthesis. *Middle:* On the left, the cell boundaries from the 1^st^, 21^st^, 41^st^, and 61^st^ fluorescent cell images are overlaid. Black markers represent the nuclear centroid positions. Red arrows indicate the migration directions of individual cells. On the right, heat map marks the distributions of cell territories in all 61 frames of the movie. The hot-to-cold color gradient illustrates the density of cell area occupancies. *Right:* A logarithm-logarithm plot of the overlapping mean squared displacement of the cells (red) and nuclei (blue) *vs*. time lag. *Inset:* one-hour cell and coupled nucleus trajectories of NIH 3T3 fibroblasts, colored in red and blue, respectively.

To test the hypothesis discussed above, we designed a barcode to display the migration information of a single cell (Fig. 1c). Movies of a single cell and its coupled nucleus were simultaneously acquired from RFP-introduced and Hoechst 33342-labeled NIH 3T3 fibroblast cultures at 1-min intervals over one hour, and were analyzed together to extract 60 *CN correlation* data points. Then, each *CN correlation* data point was assigned to a bar with a designated color based on the location of the point in the *CCD* vs. *NCD*_//_ plot, where Regions *I*, *II*, *III*, and *IV* were specified as red, yellow, blue, and green, respectively. The bars were plotted horizontally by the time of occurrence and grouped vertically by region to assemble a barcode. Following, we analyzed 50 NIH 3T3 fibroblast movies, and obtained corresponding barcodes of each movie as a barcode collection (Supplementary Information 1). Using this collection, we calculated the overall occurrence rate of each region in all 50 samples, where occurrence percentage rates of Regions *I*, *II*, *III*, and *IV* were 24%, 42%, 24% and 11%, respectively. Consequently, we selected four individual barcodes in each separate region (Fig. 2, left), which possesses significantly higher percentile of *CN correlation* occurrence than the average occurrence rate of the corresponding region, and inspected their source movies.

We analyzed these identified four cell movies (Supplementary Information 2: movie S1,2,3,4), which possess high occurrence rate of bars in a specific region, by tracing and overlaying the boundaries of the cells recorded at the 1^st^, 21^st^, 41^st^, and 61^st^ (final) frames of each movie to monitor their migration statuses (Fig. 2, center left). To illustrate the locomotion patterns better, we also used the heat map to describe the variation of the cell boundaries from all 61 imaging frames of a cell movie (Fig. 2, center right). This analysis suggests that these four cells exhibited significantly different polarity properties by their protrusions/detachments. The cell with bars significantly increasing in *Region I* migrated persistently in one direction with protrusions concentrating on the leading edge and detachments concentrating on the trailing edge. The cell with bars having more occurrence rate in *Region II* displayed less directional persistence with wider protrusions in the leading edge. The cell with bars possessing more occurrence rate in *Region III* had protrusions probe in all directions. Finally, the cell with significant bars in *Region IV* showed protrusions and detachments mainly in two distinct directions, separated in a large angle. In general, the slope of the mean square displacement (*MSD*) indexes cell motility as a function of time lag^30^,^31^ (Fig. 2, right). The slope of the *MSD* revealed that the cell with bars significantly increasing in *Region I* underwent directional migration for both the cell and nucleus. In *Region II* and *Region III*, the cell displayed diffusive movement, but the nucleus exhibited sub-diffusive movement (stationary) in the *Region III*. In *Region IV*, the cell experienced a quick directional movement again, but the nucleus displayed normal diffusive motion. Hence, the application of the *CN correlation* analysis in a period of cell movie can identify the main locomotion events contributing to the cell migration period and, as a whole, signify the overall cell migration status displayed by the individual trajectories of these cells (Fig. 2, right, *inset*).

### Evaluation of *CN correlation* analysis using a long-term single cell trajectory

**Figure 3:**
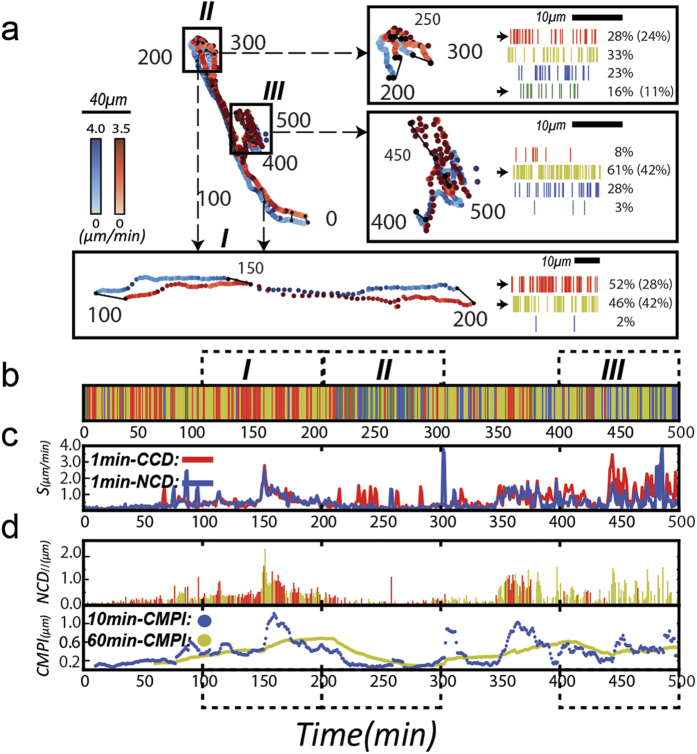
Long-term cell and nuclear trajectories and their correlations confirm the effectiveness of *CN correlation* approach in cell migration analysis. (**a**) A 500-minute cell trajectory (red dots) and its coupled nuclear trajectory (blue dots) are displayed at one-min time intervals. Dots are displayed with color gradients to indicate the instantaneous speeds. Trajectories from 100^th^ to 200^th^, 200^th^ to 300^th^, and 400^th^ to 500^th^ minutes are separately re-drawn with the corresponding barcodes. Black lines illustrate the temporal relationship between the two trajectories by connecting the cell and the corresponding nuclear positions in 50-minute time points. (**b**) Trajectories of cells are presented as a region-mixed barcode. Bars are aligned by their time sequences and each bar indicates a predominant subcellular migratory activity: detachment-dominant (red), mixed detachment and protrusion (yellow), protrusion-dominant (blue) and large-angle side protrusion (green). (**c**) Instant nucleus (blue) and cell (red) migratory speeds are shown, calculated by one-min time sequences. (**d**) Upper Row: *CN correlations* that occurred in Region *I* (red) or *II* (yellow) are depicted, where the value of *NCD*_//_ is plotted against time sequences. Bottom Row: Moving averages of *CMPI* are plotted against the time at 10-min (blue) and one-hour (yellow) intervals.

To further evaluate the *CN correlation* approach, the cell and coupled nuclear trajectories from a single NIH 3T3 fibroblast were displayed and analyzed at one-minute intervals for a 500-minute duration (Fig. 3a; also see Supplementary Information 2: movie S5). Effective cell migration events exhibited by the trajectories were generally consistent with the barcode. Again, the active migratory cell with straight trajectory had 52% of the bars in the barcode located in *Region I*, significantly greater than the average rate, 24% (Fig. 3a, Box I and the corresponding barcode). When a large-angled turning event occurred (200^th^–300^th^ min in the trajectories), the corresponding barcode contained 16% of bars in *Region IV*, relatively higher than the average rate, 11%. The presence of a higher frequency of negative *NCD*_//_ supported our previous prediction that *CN correlation* data in the *Region IV* link to large-angle side protrusions (Fig. 3a, Box II and the corresponding barcode). In fact, this would be the signature in the barcode of the cell turning event and the major difference to separate the cell turning event from the evasive cell migration event (Fig. 3a, Box III and corresponding barcode).

We further mapped the entire barcode with their instantaneous speeds. A 500-minute, region-mixed, barcode was drawn to illustrate the corresponding subcellular activities over the time course (Fig. 3b). Values of the instantaneous speed were also determined (Fig. 3c). These two charts revealed the correspondence of the detailed cell migration status. In the cases of evasive migration and turning, it can be observed that the cell momentarily moved at greater speed (red line) than the nucleus (blue line). This disparity of the speeds can correspond to bars with blue and green colors, which do not help expedite cell migration. In contrast, the comparable speeds occur when the bars are in red and yellow colors, where the cell and nucleus both moved together to promote cell migration.

These results are consistent with the prediction from the *CN correlation* analysis. In active cell motion, in which bars are distributed significantly in *Region I* and *II*, the cells and nuclei would have a strong, alternative corresponding locomotion. In cell turning events, the cells start with a large angle protrusion, followed with the nuclear direction change. In the evasive motion events, cells have less corresponding movements between the cells and nuclei with considerably more side protrusions, compared to the active motion.

### Cell Migration potential index (*CMPI*) predicts mesenchymal-like cell motility

Based on the signatures of the subcellular migratory activities represented in the four regions of the *CCD-NCD*_//_ coordinate system, we postulated that relative motility of mesenchymal-like cells could be gauged by a cell migration potential index (*CMPI*) according to the equation:


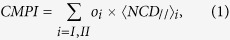


where *o*_*i*_ denotes the occurrence of *CN correlation*s over populations of the same cell type in a specific *region*_*i*_, <*NCD*_//_>_*i*_ represents the mean value of *NCD*_//_ over populations of the same cell type in the specific region, and the subscript *i* represents either Region *I* or *II* in the *CCD*-*NCD*_//_ coordinate system. According to this definition, a greater *CMPI* value of a cell should indicate a greater motility.

The *CN correlations* identify effective cell movements by extracting significant nuclear translocations in the moving direction of the cells, which are located in the Region *I* and *II* of the coordinate system. In contrast, nuclear translocations in Region *III* or *IV* do not directly contribute to cell motility and can be ignored. The *CMPI* was designed to capture the effective nuclear movement events, represented by the *NCD*_//_ shown in Region *I* and *II* (red and yellow, respectively) over the time course (Fig. 3d, *top row*). Once those *NCD*_//_ values were accumulated as an index for cell motility, the short-term running *CMPI* (*e.g*., 10 minutes here) can reveal the migration details of the cells, whereas the long-term running *CMPI (e.g*., 60 minutes here) approach a stable value that might represent the overall cell motility (Fig. 3d, *bottom row*).

Taken together, *CN correlation* analysis might assess cell motility in real time and, as the analysis shows, each *CN correlation* datum can represent the corresponding subcellular locomotion mode.

### Evaluation of *CMPI* using the Persistent Random Walk (*PRW*) model

**Figure 4:**
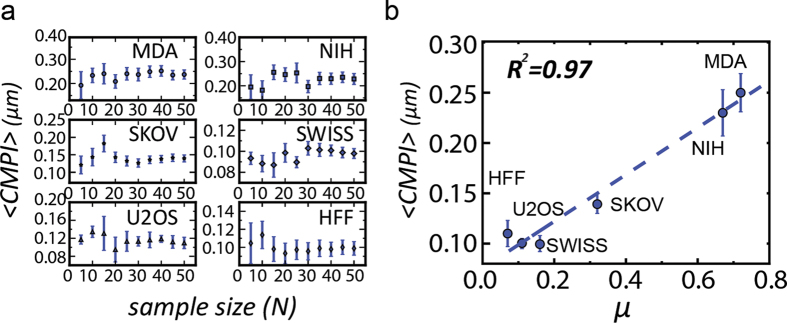
A sample size of 20 one-hour cell migration movies is sufficient to derive precise and accurate *CMPI* for cell motility description. (**a**) Means and variances of *CMPI* are plotted against sample size for 6 different types of cells. Error bars represent standard error of the mean. (**b**) Correlation between *CMPIs* and diffusion coefficients for 6 different cell types. A blue dot represents a cell type, and the dashed line is the fitting curve. Abbreviations for cell types: MDA-MB-231 (MDA), NIH 3T3 (NIH), SKOV-3 (SKOV), OVCAR-3 (OVCAR), U-2 OS (U2OS), SWISS 3T3 (SWISS) and Human Foreskin Fibroblasts (HFF).

We hypothesized that the averaged *CMPI* value (expressed as <*CMPI*>) from multiple cells of a cell type might be used to represent the motility of the cell type. To test this hypothesis, we first evaluated the precision of <*CMPI*> against the cell sample size. The cell types studied included 3 cancer cell lines: MDA-MB-231, SKOV-3, and U-2 OS cells, and 3 fibroblast lines: NIH 3T3, Swiss 3T3, and human foreskin fibroblasts. <*CMPI*> of each cell type was calculated from different cell sample sizes. At each iteration, samples of each cell type were independently and randomly chosen from a pool of 50 samples and cell sample size was increased by a step of 5. At small sample sizes (1-min time intervals and one-hour monitoring time), the <*CMPI*> of all six cell types fluctuated. However, when the sample size exceeded 20, the <*CMPI*> approached a steady state, in which the standard error of the mean diminished to the confidence limit (less than 10% of the mean value) (Fig. 4a). These results indicate that a stable <*CMPI*>, *i.e*., exhibiting a good convergent tendency, can be obtained from a relatively small cell sample size (~20).

The <*CMPI*> and standard errors of each cell type were calculated from data extracted from 50 one-hour cells and coupled nuclei movies, acquired at one-min time intervals, to verify the accuracy of *CMPI* in describing the trends of cell motility among different cells (Table 1). These data were compared to their corresponding diffusion coefficients, *μ*, obtained from the Persistent Random Walk (*PRW*) model^17^,^32^,^33^. The diffusion coefficient of each cell type was extracted from ~15 nuclear trajectories, tracked at 1-min time intervals for 10 hours. The fitting between the diffusion coefficient and *CMPI* produced an *R*^*2*^-value of 0.97 (Fig. 4b). Hence, this result demonstrates that *CMPI* provides an excellent estimate of the motility of mesenchymal-like cell types, and indicates that the motility of single mesenchymal-like cells can be efficiently described at short time intervals under the *CN correlation* approach using the cell migration potential index, *CMPI*.

### The impact of positioning error in the subcellular activity classification

**Figure 5:**
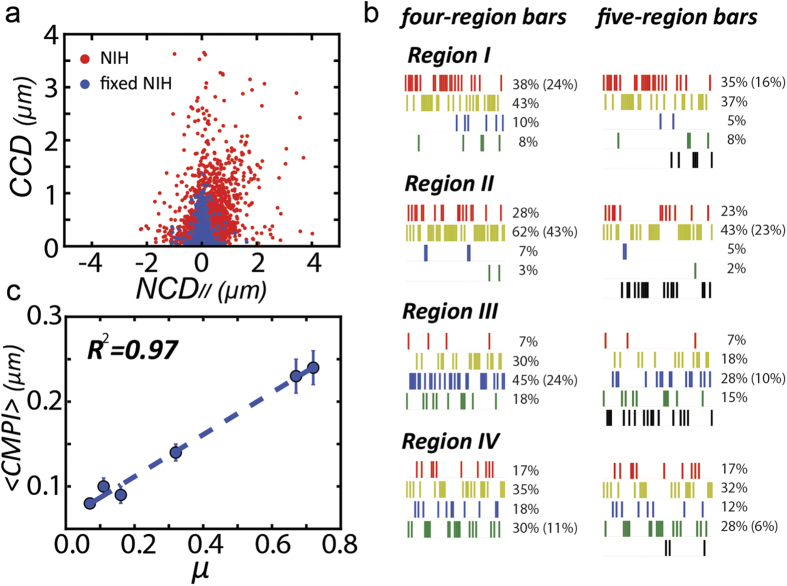
*CMPI* estimates are independent of positioning errors. **(a)** *CCD* and coupled *NCD*_//_ of 50 single live NIH cells (red dot) and fixed NIH cells (blue dots) in the *CCD vs*. *NCD*_//_ plot. **(b)** *Left:* Original barcode of an individual NIH cell tracked at 1-min lag time for 60 min. *Right:* Error-filtered barcode of the same tracking data. **(c)** Linear relationship of diffusion coefficient and error-filtered *CMPI*.

When acquired from shorter time intervals, the magnitudes of the *CCDs* and *NCDs* inevitably become smaller. Hence, the imaging acquisition errors will have more significant impacts to the data. To evaluate this impact, a fixed-cell was subjected to the *CN correlation* analysis to obtain the positioning error profile in the *CCD-NCD*_//_ coordinate system. Since the probed cell was fixed, the non-zero distribution of its *CN correlation* data in the *CCD vs. NCD*_//_ plot was merely contributed from the acquisition errors, which formed a special region in the *CCD-NCD*_//_ coordinate system that could be treated as a separate distribution, called *Region V*: *Stationary Mode*. The results confirmed that the positioning error cannot be ignored (Fig. 5a). However, it cannot be an implication that every *CN correlation* datum located within the zone is in stationary mode since the positioning error could also make a non-zero *CN correlation* datum shift and look “stationary”.

Hence, we used the Monte Carlo method to create a probability density function by the distribution of the fixed-cell data, denoted as *p*(*NCD*_//_,*CCD*), to evaluate whether a *CN correlation* datum generated from a live-cell belongs to the stationary mode. Afterwards, we randomly generated decimals in range [0, 1] for each live-cell datum (denoted as subscript *i*). If this random value is greater than the noise probability, *p*_*i*_(*NCD*_//_,*CCD*), the datum is treated as an effective motion mode. If the random value exhibits a converse property, it will be considered as an ineffective motion mode, as shown below.


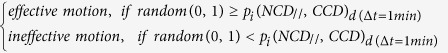


Through this approach, whether a datum belongs to the stationary mode can be identified.

We further evaluate whether the previous *CMPI* estimate might be biased without considering the *CN correlation* data in the stationary mode. We defined a new fifth row in the barcode as *Region V*, in which the bars are labeled in black. We then re-probed cells, and shifted those ineffective subcellular modes from other regions into *Region V* (Fig. 5b). After using the five-region barcode for *CN correlation* analysis, we continued comparing the new *CMPI* values and the diffusion coefficients derived from the *PRW* model in six mesenchymal-like cell lines. The linear fitting has an *R*^*2*^ value = 0.97 (Fig. 5c, detailed data see Supplementary Information 3), which is comparable to that from the four-region barcode. Hence, this practice supported that the absorption of those insignificant stationary modes into the four-region barcode will not impact the *CMPI* estimate to describe the motility trends among different mesenchymal-like cell lines. In addition, unless a cell stays in the stationary mode for a prolonged time (*e.g*., more than five minutes), we may not consider the cell as being in the stationary state. When cells probed in such a short time lags (*e.g*., 1-min), a normal locomotion event could have a certain discrete period (1–3 minutes) showing in the stationary mode.

### Robustness of *CMPI* analysis

To obtain correct *CMPI*, we applied fixed particles to the glass-bottom dishes as reference points among image frames to minimize positioning errors generated during routine computer-controlled data acquisition using a particle tracking technique^34^. We also developed a Gaussian filter algorithm to identify the cell boundary with a level of accuracy that cannot be achieved using the normal segmentation process^35^. Consequently, the errors generated during image acquisition and analysis could be greatly reduced. Nonetheless, intrinsic errors are still present during image acquisition. These intrinsic errors may obscure the true displacement of cells or nuclei if the observation time intervals are too short. However, if the observation period is too long, individual subcellular activities would be overlooked, again generating inaccurate estimates of *CMPI*. Hence, an optimal temporal setting is needed to be set for the documentation of individual subcellular activities. Therefore, a series of robustness tests were applied to determine the appropriate time intervals for image documentation and to examine the effects of intrinsic errors (noise) on *CMPI*.

**Figure 6:**
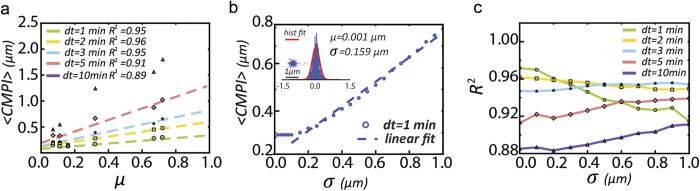
One to three-minute time intervals can describe the robust *CMPI* utilizing various image acquisition systems. **(a)** *CMPI* of 6 different cell types calculated at 1, 2, 3, 5, or 10 min time intervals are compared against the corresponding diffusion coefficients, *μ*. **(b)** Random Gaussian noise-implemented *CMPI* values are plotted against the standard deviation of the Gaussian noise (ranging from 0–1.0 μm). The initial data were obtained from NIH 3T3 fibroblasts at 1-min time intervals. *Inset*: The distribution of system noise was determined from the centroid positions of a fixed cell. **(c)** The fitting results (*R*^2^) between noise-implemented *CMPI* and corresponding diffusion coefficients, *μ*, of 6 cell types were plotted against the standard deviation of introduced noise (ranging from 0–1.0 μm). *CMPI* were calculated using data extracted at 1-, 2-, 3-, 5-, and 10-min intervals from 1-hour movies of the 6 different cell types.

To determine the optimal time intervals at which to record the *CMPI*, the *CMPI* of the 6 mesenchymal-like cell types were calculated at different time intervals, τ = 1, 2, 3, 5, and 10 min, from 50 cells during a one-hour monitoring time. The resultant *CMPI* were linearly fitted to the corresponding *PRW*-derived diffusion coefficients (τ = 1 min) to determine the time interval at which the derived *CMPI* most closely correlated with the motility trends of different cells acquired via the *PRW* model. The results showed that the most consistent trends between the *CMPI* and their corresponding diffusion coefficients (with an *R*^*2*^ value ≥ 0.95) occurred at time intervals of 1 to 3 minutes (Fig. 6a).

We then evaluated the impact of the intrinsic system noise on the estimation of *CMPI*. To do so, we first determined the contribution of intrinsic errors (*i.e*., the uncontrollable errors) generated in our microscopy system. Since the correctable errors generated from the image acquisition and analysis process had been minimized by fixed particles, we attributed the remaining factors that resulted in inconsistent centroid positions of a fixed cell to intrinsic system errors. We repeatedly measured the centroid positions of fixed cells to obtain the distribution and variance of the centroid positions, which displayed as a Gaussian distribution in both x- and y-coordinates with the mean at the center and the standard deviation (σ) = 0.17 μm and 0.16 μm, respectively (Fig. 6b, *inset*). Here, the standard deviation of the distributions (~0.2 μm) can be treated as the intrinsic errors of our microscopic systems.

We assessed the impact of the intrinsic system noise on the estimation of *CMPI*. To approach this, a randomly generated Gaussian noise (with the mean = 0 and standard deviation = σ) was applied to each of the experimental *CCD* and *NCD* data points of NIH 3T3 fibroblasts to distort the displacement measurements (for this analysis, the experimental data were assumed not to possess any errors).


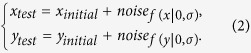


After this procedure, an error-implemented *CMPI* was calculated. The resulting <*CMPI*> values, obtained from 1000 repeats of noise-implemented *CMPI* determinations at a given σ (ranging from 0.02 to 1 μm), showed that the system noise had no significant impact on the <*CMPI*> values when σ was less than 0.15 μm but exhibited a linear correlation as σ increased beyond 0.15 μm (Fig. 6b). This result suggests that <*CMPI*> determinations are influenced by the intrinsic system errors in a linear manner when the image acquisition system possesses an intrinsic system error greater than 0.15 μm.

Since the *CMPI* provides relative motility among different cell types, the relevant issue is whether different acquisition systems (with different intrinsic system errors) will generate very different *CMPI* results that would affect the comparison of cell motility among cells. Hence, we further assessed the competence for using *CMPI* to judge the relative motility among different cell types in the presence of different sizes of system errors. The noise-implemented <*CMPI*> of the 6 cell types were then calculated at different σ values (ranging from 0 to 1 μm) at various time intervals (1, 2, 3, 5, and 10 min) and linearly fitted to the corresponding diffusion coefficients. The results indicate that the *CMPI* calculated from τ = 1–3 min accurately described motility trends (*R*^2^ ~ 0.96) if the system noise (σ) was <0.3 μm. *CMPI* calculated from τ = 2–3 min can consistently describe motility trends when the noise was between 0.3 and 0.8 μm (Fig. 6c).

The study presented here demonstrates that the *CN correlation* approach can be used to accurately and reliably estimate cell motility. *CMPI* is a robust index of cell motility for various cell types, and is resistant to intrinsic noise carried by a normal microscopic system. Hence, *CMPI* is a biologically relevant index for mesenchymal-like cells, linking cell motility to corresponding subcellular activities.

## Discussion

In this study, we introduce a novel *CN correlation* approach to analyzing cell migration, in which the motion of the cell relative to the motion of the nucleus is utilized to classify and weight the stages of cell migration into four momentary subcellular events: (1) detachment dominant, (2) mixed detachment and protrusion, (3) protrusion dominant, and (4) large-angle side protrusion. We adopt a barcode system to assist in the visualization of dynamic processes and their temporal occurrence in single cells. We also define a quantitative cell migration potential index (*CMPI*) to characterize these subcellular activities. Using *CMPI*, cell migration can be deciphered in detail, and cell motility can be delineated according to discrete biological events. This approach provides an avenue to connect cell migration to its underlying subcellular activities, explains the effects of associated molecular events, and further delivers a powerful tool to associate individual molecular mechanisms with their ensemble cellular activities.

Reliable cellular measurements are essential to the *CN correlation* approach. Identification of correct cell boundaries, including thin lamellipodia, to determine centroid information during imaging analysis is a prerequisite procedure. It is vital to select appropriate time intervals that not only support highly resolved individual subcellular documentation, but also distinguish cell and nuclear displacements from background noise. In previous studies^34^,^35^, we provided detailed imaging processing guidelines to ensure successful *CN correlation* analysis. Since we adopt short time intervals in this study, the accuracy of boundary information is critical. The short time intervals further fragment the subcellular locomotion events into smaller subevents, in which the displacements between the time frame could be largely affected by the errors generated through the imaging acquisition and boundary determination. Hence, a real stationary mode cannot be easily separated from small motion. We therefore provide a Monte Carlo approach and use a five-region barcode to separate the stationary modes from small motions to determine the impact of the stationary modes. Since these small motions are more likely to appear in *Region II* and *Region III*, identification of active motions from subevents would not be affected much; hence, we mainly adopt the four-region barcode in this study to build up the migration capability index. This choice has been demonstrated to preserve the integrity of the cell migration states and retain cell motility trends accurately among different cell lines. In case the stationary state needs to be specifically identified for a certain purpose, *Region V* can be applied to the barcode to isolate continuous stationary modes.

In this work, we develop *CMPI* and demonstrate the reliability and adaptability of *CMPI*. The cells often move in the 3-dimensional (3-D) environment under physiological conditions, in which the physiological morphology and stiffness of the extracellular matrix vary broadly to affect cell motility. Hence, it would be difficult to claim a reliable quantitative method to link subcellular activities and assess cell motility across various environmental conditions. On the other hand, the *CN correlation* approach introduced here can smoothly deliver a precise, quantitative description to link subcellular activities (therefore underlying signaling pathways) to cell motility for fundamental biomedical study and the pharmaceutical industry.

Currently, the wound-healing assay and the *PRW* model are the two main methods used for comparison of migration potentials among related cell types. The wound-healing assay is a straightforward method to assess the capacity of cells grown to repopulate (heal) a mechanically introduced cell-free zone through collective cell migration and proliferation. In the wound-healing assay, cell migration (and/or proliferation) is quantified as the void area occupied by cells. The *PRW* model, on the other hand, treats individual cells as particles, and utilizes their mean squared displacements (*MSDs*) to characterize cell motility. Through combining these two cell migration assessments with molecular manipulation, our understanding of cell migration at both cellular and molecular levels has greatly advanced. However, in both of these systems, biophysical information that details the subcellular events, which guide distinct cell migration patterns and account for en route cell motility changes, are not assessed directly. The *CN correlation* approach can comprehensively incorporate the subcellular events into cell migration analysis.

In this study, we use *CMPI* to analyze and compare the trends of six cell types. We show that the motility trends of the six cell types overall determined from *CMPI* and *PWR* are highly consistent, with *R*^*2*^ > 0.95. Since the *PWR* model is derived from a mathematic approach, several specific assumptions regarding cell migration have been proposed in this model. These assumptions include instantaneous speed being time-independent, Gaussian distribution of velocities, exponential decay for the autocorrelation function, and isotropic cell movement. However, these assumptions cannot be always satisfied for different cell types due to heterogeneous cell behaviors. Therefore, the mathematical procedures need to be constantly modified to match varying cell migratory features^36^,^37^. In contrast, the *CMPI* approach requires no prior assumptions, but is directly rooted in the physiological behavior of the cells with straightforward mechanical analysis.

The *CN correlation* and *PRW* model both utilize trajectory information to realize mesenchymal-like cell migration. The *PRW* model utilizes speed (*S*^2^) and persistence time (*P*) as two main parameters to determine cell migration capacity. For accurate migration analyses, a long tracking time (~10 hrs) is required. The occurrence of photo-bleaching from extensive light exposure during the tracking period can interfere with the analysis. In contrast, the *CN correlation* approach analyzes migratory behavior of individual cells over a relatively short monitoring time (e.g., 1 hour), and requires small sample sizes (approximately 20 cells). Therefore, the assay conditions are more favorable toward reducing photobleaching effects and experimental time.

In addition, *CMPI* is not limited to describing the motility of a mesenchymal cell type; it should also be used to describe the dynamic processes of any individual cell in real time. Every protrusion or contraction event can be separately and sequentially recorded in a barcode. When a barcode is applied to describe the migratory process of a single cell, the barcode and *CMPI* can facilitate the visualization of the individual cell migration process in a digitized computer format. However, the *CN correlation* approach can be exploited. For example, in analyzing kinetic processes during drug therapy, this approach can provide information regarding the impact of a drug on subcellular and cellular processes during cell motion as a cell dynamic assessment method for applications such as clinical trials.

Based on the presenting capacity of the *CN correlation* approach to mapping different subcellular activities in a cell migration event, we further provide the autocorrelation information among the *CN correlation* data. CN correlation contains two types of information: angular values (to identify what type of locomotion) and *NCD*_//_ (to indicate nuclear speed) through the *CCD*-*NCD*_//_ coordinate system. We conduct the autocorrelation among these two types of information by three time intervals (1–3 min) and in the four different significant locomotion cases showed in Fig. 2. The results of the angular autocorrelations indicate whether a temporal pattern exists within a cell locomotion event while the *NCD*_//_ autocorrelations evaluate the consistence of the nuclear speed or a specific repeated pattern is present. The results (Supplementary Information 4) confirmed that clear NCD_//_ autocorrelations exist in all four cell locomotion cases, which verifies the availability of *CN correlation* analysis. In opposite, the angular autocorrelations are fuzzy, mainly owing to its nonlinear nature in describing the subevents of the locomotion. Although these two types of autocorrelations are seemingly disjoint to each other, they should have a certain type of connection through the *NCD*_//_ values (*i.e*., the *NCD*_//_ should possess inertial to prevent an abrupt change in cell locomotion). Thus, we will study the connection between these two types of autocorrelations to recognize migration patterns in the future work. In addition, 1–3 min time interval analysis results also have a consistent correlation trends, supporting the validity of *CN correlation* data at different time analysis scales.

In conclusion, wound healing scratch assays may be better for high throughput, whereas *CMPI* would be better for studying cancer-related EMT gene expression, etc. wherein the molecular mechanisms are important. The results of this study provide an effective and efficient means to analyze mesenchymal-like cell migration.

## Methods

### Cell cultures, plasmid transfection, and nuclear stain

MDA-MB-231, NIH 3T3, and Swiss 3T3 cell lines and human foreskin fibroblasts (all purchased from ATCC, Manassas, VA) were cultured in DMEM containing 10% fetal bovine serum (FBS) and 1% L-glutamine (Mediatech). U-2 OS cells (ATCC) were cultured in McCoy’s 5A medium with 10% FBS and 1% L-glutamine (Mediatech). Cultured cells were maintained in a humidified incubator at 37 °C and 10% CO_2_. pRFP-R-CS plasmids (Origene, Rockville, MD), containing red fluorescence protein (RFP) gene, were introduced into cells using Lipofectamine 2000 (Invitrogen, Carlsbad, CA) following the standard protocol. Subsequently, the cells were seeded on fibronectin (BD Biosciences, San Jose, CA)-coated glass bottom dishes for 24 hours prior to image acquisition. Live-cell nuclear stain, Hoechst 33342 (Sigma-Aldrich, St. Louis, MO), was added to the medium 10 minutes prior to image acquisition.

### Microscopy

A Nikon TE-2000 microscope (Nikon, Melville, NY), equipped with an X-Cite 120PC fluorescent light source (EXFO, Ontario, Canada), a Cascade: 1 K charge coupled digital camera (Roper Scientific, Tucson, AZ), and an on-stage incubator with CO_2_ supplementary system (*In Vivo* Scientific, St. Louis, MO), was used to acquire images of cell and nucleus motion. The image acquisition environment was kept at 10% CO_2_ and 37 °C during experiments.

The fluorescent images of cells and the concomitant nuclei were sequentially acquired using a 20× objective lens (Nikon) at a frequency of one-minute per acquisition cycle. For cell migration potential index (*CMPI*) determination, movements of cell and nucleus were acquired over one hour in general case. An acquisition over 500 minutes was also applied for long-term analysis. For persistence time calculation, the nuclear trajectories were acquired over ten hours. Microscopy parameters were 100 ms exposure time, 3 × 3 bin size, and 25% power of light source.

### Single cell trajectory analysis

Cell and nucleus centroids were determined from the sequentially acquired images based on segmentation performed on each image frame. Subtle changes in cell morphology were faithfully captured using our previously developed spatial filtering scheme^35^. The measured centroid can accurately reflect those changes, enhancing the spatial resolution of cell and nucleus trajectories. A trajectory was determined by linking the geometric centers of the tracked object through the time sequence. The long-time (10-hour) nuclear trajectories served as the source information to calculate persistence time using a custom-made program in MATLAB (Mathworks, Natick, MA). The persistence time of each cell type was determined by fitting the mean square displacement, 〈*r*^2^〉, with root mean square speed, *S*, using the persistent random walk equation: 〈*r*^2^〉 = 2*S*^2^*P*[*t*  −  *P*(1 − *e*^−*t*/*P*^)], where *P* and *t* are the persistence time and time interval, respectively^38^,^39^. We adopted Levenberg-Marquardt algorithm to perform the least square fitting since it gave the best fitting results for our cases. The diffusion coefficient, *μ* = 〈*S*^2^〉*P*/*n*, where *n* (=2 here) is the dimension of extracellular space, was derived from *P* and 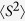
17.

## Additional Information

**How to cite this article**: Lan, T. *et al*. Displacement correlations between a single mesenchymal-like cell and its nucleus effectively link subcellular activities and motility in cell migration analysis. *Sci. Rep*. **6**, 34047; doi: 10.1038/srep34047 (2016).

## Supplementary Material

Supplementary Information

Supplementary Movie S1

Supplementary Movie S2

Supplementary Movie S3

Supplementary Movie S4

Supplementary Movie S5

## Figures and Tables

**Table 1:**
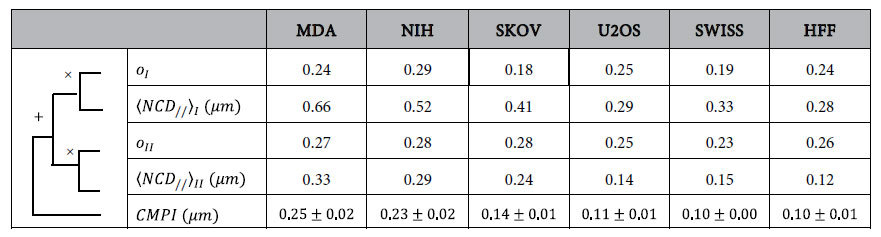
Cell migration potential index (*CMPI*) from 6 different cell types.

*CMPI* values were calculated according to the equation: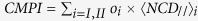. Input data were acquired at one-min time intervals from one-hour movies. N = 50 cells.

Values are reported as *mean* ±  *SE* (*standard error*) from 50 cells.

MDA, NIH, SKOV, OVCAR, U2OS, SWISS, HFF is the abbreviation of cell lines MDA-MB-231, NIH 3T3, SKOV-3, OVCAR-3, U-2 OS, SWISS 3T3 and Human Foreskin Fibroblast, respectively.
