# Supplementary material for: Displacement correlations between a single mesenchymal-like cell and its nucleus effectively link subcellular activities and motility in cell migration analysis
Source: Sci Rep. 2016 Sep 27;6:34047. doi: 10.1038/srep34047 (PMC5037420; doi:10.1038/srep34047)
Supplement: Supplementary Information [file srep34047-s1.pdf]

Displacement correlations between a single mesenchymal-like cell and its nucleus effectively link subcellular activities and motility in cell migration analysis

Tian Lan[1], Kai Cheng[1], Tina Ren[4], Stephen Hugo Arce[1], and Yiider Tseng[1,2,3,5,†]

[1]Department of Chemical Engineering, [2]J. Crayton Pruitt Family Department of Biomedical Engineering and [3]Institute for Cell & Tissue Science and Engineering, University of Florida, Gainesville, FL 32611, USA, [4]Harvard School of Dental Medicine, Boston, MA 02115, USA. and [5]National Cancer Institute-Physical Science Oncology Center, Gainesville, FL 32611, USA.

[†]Address correspondence to:

Yiider Tseng, Ph.D.
Chemical Engineering Building, Room 223
1006 Central Drive
University of Florida
Gainesville, FL 32611-6005
Tel: (352) 392-0862
Fax: (352) 392-9513
Email: ytseng@che.ufl.edu

SUPPLEMENTARY INFORMATION

Supplementary information 1:

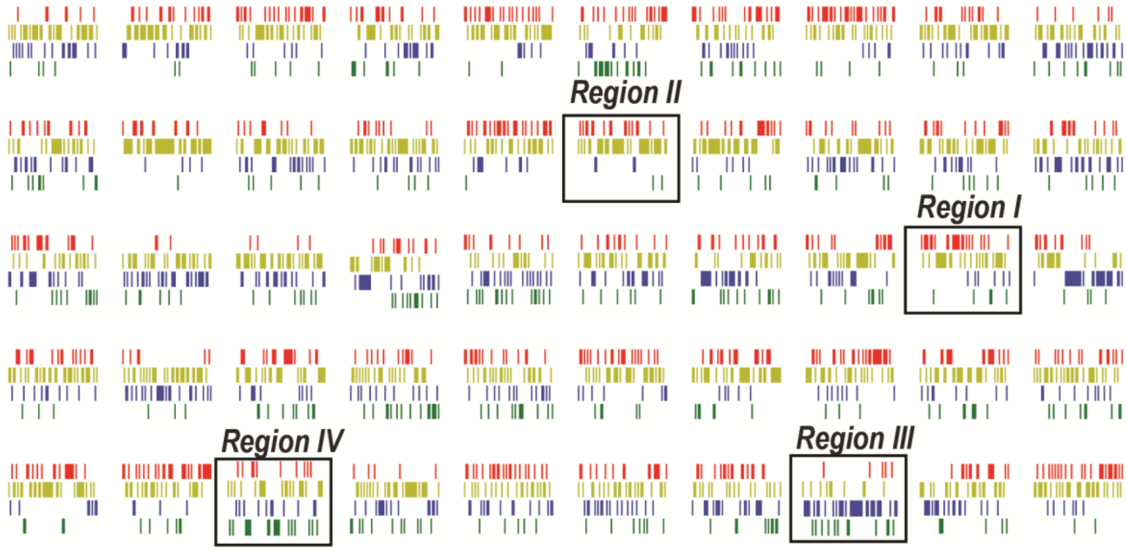

Figure S1. One-hour migration movies of 50 cells were analyzed using *CN-correlation* barcodes to document their migration patterns. Each barcode represents the migration pattern of a randomly selected individual NIH 3T3 fibroblast (RFP-tagged) at one-minute intervals over a one-hour period. Three barcodes are marked as examples of barcodes dominated by a type of subcellular activity in the *CN correlation* (*Region I, II*, *III*, and *IV* respectively). Four movies (Movie S1-4) correspond to these barcodes. B. The four selected barcodes are chosen based on the most significant occurrence increase of the bar in a specific region (see the main text for explanation).

Supplementary information 2:

Movie S1-S4. Each movie displays a typical cell migration mode in the *CCD*-$NCD_{//}$ coordinate system as *Region I*, *II, III* and *IV*, respectively. The corresponding barcodes were identified in Figure S1. Each movie records migration of a single RFP-tagged NIH 3T3 fibroblast at 1-min intervals over a one-hour period.

Movie S5. The movie of a single RFP-tagged NIH 3T3 fibroblast was acquired at 1-min intervals over a 500-minute period. This movie was analyzed in Figure 3.

Supplementary Information 3:

	MDA	NIH	SKOV	U2OS	SWISS	HFF
o_I	0.24	0.28	0.17	0.17	0.18	0.23
$\langle NCD_{//}\rangle_I\ (\mu m)$	0.67	0.52	0.46	0.42	0.34	0.31
o_{II}	0.25	0.26	0.25	0.26	0.12	0.22
$\langle NCD_{//}\rangle_{II}\ (\mu m)$	0.34	0.29	0.25	0.25	0.16	0.15
$CMPI\ (\mu m)$	0.24 ± 0.02	0.23 ± 0.02	0.14 ± 0.01	0.10 ± 0.01	0.08 ± 0.00	0.09 ± 0.01

Table S1. Error filtered cell migration potential index (CMPI) of 6 different cell types.

Supplementary Information 4:

Autocorrelation analysis of *CN correlation* reveals four different cell migratory patterns

We have demonstrated that each *CN correlation* datum can be mapped to one of the four specific locomotion modes through its location in the *CCD vs.* $NCD_{//}$ plot using the polar coordinate system. Hence, we obtained *CN correlation* data from a one-hour cell movie in which the cell performed a distinctive locomotion event. The angular coordinate and the magnitude of the $NCD_{//}$ of the *CN correlation* data were subjected to autocorrelation analysis to understand whether temporal patterns exist in these two variables during different locomotion events. In the analysis process, the autocorrelation coefficients were computed through using overlapping time intervals, while the *CN correlation* data were obtained at various time intervals, ranging from one to three minutes.

The autocorrelation of $NCD_{//}$ displayed reasonable results in accordance with their corresponding migration patterns. An active migratory cell showed relatively strong autocorrelation with adjacent time lags. This is expected since during active migration the nucleus should routinely perform translocations with the repeated trailing edge detachments. According to the $NCD_{//}$ autocorrelation result, the nucleus can maintain a relatively similar velocity for approximately three to four minutes, *i.e.*, even though the nucleus might still move in the same direction after four minutes, the nuclear speed can display a significant difference. For an evasive migratory cell, the $NCD_{//}$ autocorrelation was dramatically decreased and the result suggested that there is no clear temporal migratory pattern. A cell undergoing evasive motions displays less persistence to the direction in respect to the active migration. Although the inertia (displaying as the *NCD*) to resist change from the previous movement plays a critical role in nuclei movement, the magnitude of the $NCD_{//}$ could abruptly change once the cell changes its direction. A sampling cell, on the other hand, has a stationary nucleus ($NCD_{//} \sim 0$) regardless of its protrusions occurring in random directions. Hence, the correlogram had random and vigorous oscillation around 0. A

cell with a large-angle protrusion also possessed a largely fluctuating autocorrelation from its *CN correlation* data as the sampling cells, while displaying obvious autocorrelations when the *CN correlation* data were obtained at time intervals of 3 minutes. This result can be associated with the cell turning pattern. When a cell proceeds in a large-angle turning event, the inertia of nuclear motion occurs in a regular pattern that can be sustained and observed at the sixth minute (autocorrelation was dropped after six minutes). However, this autocorrelation only can be observed in longer time intervals (3 minutes) in respect to the shorter time intervals (one to two minutes). This is presumably because the continual detachment events, due to cell polarity changes, have a frequency in between two and three minutes and the nuclear motion is controlled by the detachment events. Hence, nuclear motion cannot be smoothly detected in the shorter time intervals.

In contrast to the $NCD_{//}$ autocorrelations, the angular autocorrelations among the four types of migration activities are similar to each other. The autocorrelations decay quickly in different analyses of time intervals, indicating that the angular patterns have no regular trends. Indeed, a cell locomotion event is assembled by various locomotion modes, which can take place without any special order, and it certainly does not require that the component modes consist of a set pattern.

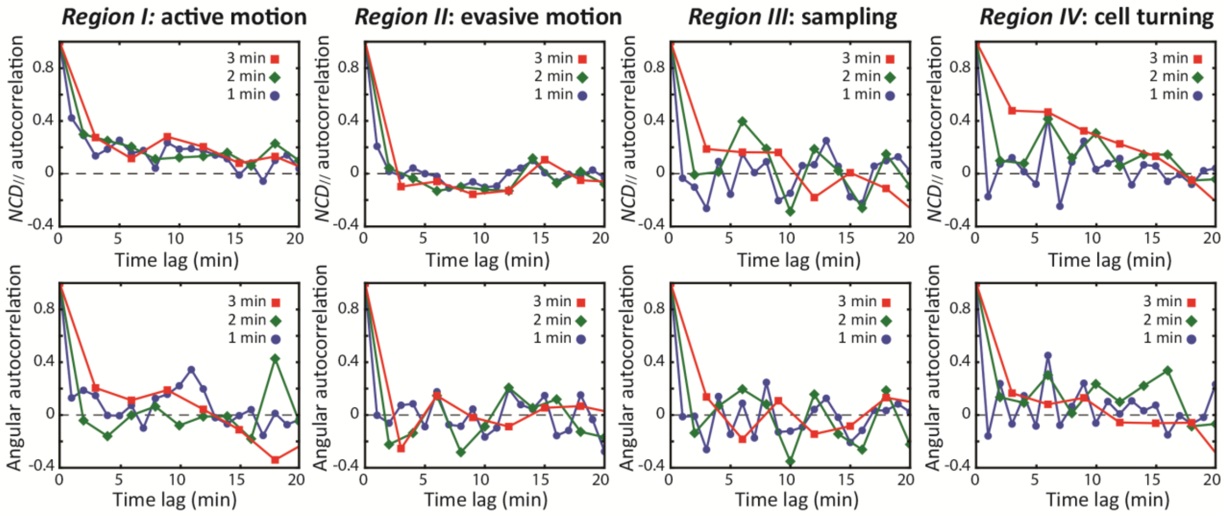

Figure S2. Autocorrelations of the *CN correlation* data representing a specific migratory activity. The $NCD_{//}$ autocorrelation (top) and the angular coordinate autocorrelation (bottom) are calculated from *CN correlation* data extracted from one-hour cell movies in which the cells are undergoing a specific cell migratory activity. Dots display the autocorrelation results at τ = 1 (blue), 2 (green) and 3 (red) minutes overlapping time intervals, respectively.
